# Prespecified analysis plans are not suitable for exploratory experimental physiology research

**DOI:** 10.1113/EP093575

**Published:** 2026-02-12

**Authors:** Erik A. Richter, Bente Kiens, Abigail L. Mackey

**Affiliations:** ^1^ Section of Human and Molecular Physiology, Department of Nutrition, Exercise and Sports, Faculty of Science University of Copenhagen Copenhagen Denmark; ^2^ Institute of Sports Medicine Copenhagen, Department of Orthopedic Surgery Copenhagen University Hospital – Bispebjerg and Frederiksberg Copenhagen Denmark; ^3^ Department of Clinical Medicine, Faculty of Health and Medical Sciences University of Copenhagen Copenhagen Denmark

**Keywords:** exploratory, physiology, preregistration, science

1

A recent editorial in Experimental Physiology entitled ‘Role of prespecified analysis plans in physiological research: Encouraged or mandatory?’ (Christensen et al., 2025) is an eye‐opening warning of another potential slide down the regulatory pathway in science. If implemented, as the authors suggest, it will in our view kill exploratory experimental studies in humans – and for that matter also in animal models and cells. This is just another inappropriate regulatory burden in addition to the requirement for pre‐registering basic human experimental trials in a clinical trials database that several journals require. This is despite the obvious misconception classifying exploratory physiological experiments as clinical trials (Richter et al., 2024).

Christensen et al. write that ‘A detailed prespecified statistical plan is a prerequisite for ensuring the validity, reliability and replicability of study findings, as it is essential for upholding methodological rigor.’ This may be true for clinical studies but not for exploratory physiology studies. A prespecified statistical plan is no guarantee for a good study. We can ask: why do you perform research? Is it to fill data into a detailed prespecified statistical plan which you can only do if you know what kind of outcome the study will produce? This might be fine if you are studying the potential effect of a drug or a certain clinical procedure or want to confirm a previous study, but if you are performing experimental exploratory basic physiology the outcome is and should be unpredictable. Such studies cannot be boxed in rigid preregistrations and in rigid predefined statistical analysis plans. Experimental basic exploratory physiology is carried out to explain basic fundamental, often molecular mechanism and are mainly hypothesis generating rather than hypothesis driven. Thus, it is important to be able to distinguish *de facto* clinical trials where preregistration and prespecified statistical plans are sensible, from basic experimental exploratory experiments where the outcome is unknown. Experimental physiological studies form the knowledge base for later clinical trials. Without experimental studies, future breakthroughs in medical treatment will be scarce. What characterizes a great basic exploratory study is not a prespecified statistical plan but great innovative ideas, appropriate state of the art technology, trained personnel, a well‐designed protocol, and an open and honest mind to discover novel mechanisms.

An underlying argument of the editorial by Christensen et al. seems to be the assumption that the general practice of experimental researchers is to ‘rationalize biased *post hoc* omissions and spin’, through ‘solely focusing on statistically significant or deemed “interesting” analyses’. The editorial thus implies that researchers are generally biased in presentation of their results, which is quite offensive given the many researchers that work hard and honestly to produce the best research possible.

Christensen et al. further argue that ‘Our advocacy for prespecified plans in basic experimental science should be seen as an attempt to mitigate the broader, well‐documented reproducibility crisis in the life sciences’. While we agree that lack of reproducibility is a problem, a prespecified statistical analysis plan is not going to solve that problem because it is not limited to basic science but also occurs in clinical trials where preregistration is a must (Van Noorden, 2023). In the Van Noorden paper, it is estimated that in some clinical fields, at least one‐quarter of clinical trials might be problematic or even entirely made up despite preregistration in a clinical trials database. Thus, preregistration in one form or the other does not solve the reproducibility problem and the only thing a meticulously pre‐specified statistical plan guarantees is that the study will produce predictable outcomes and not discoveries worthy of a Nobel Prize! The reproducibility issue is much better tackled by asking researchers to reproduce their novel findings in another cohort or in another setup in the same study.

Related to pre‐specified statistical plans, *Experimental Physiology* offers potential authors to submit a preregistration plan (Rasmussen et al., 2025). ‘Preregistration involves detailing and publishing a research plan, including hypotheses, design and analysis, before data collection begins.’ Some of the listed benefits are that ‘It helps to avoid improper research practices, such as choosing outcomes based on results or making hypotheses after seeing results.’ This might be fine for inexperienced researchers or for confirmatory research, but in practice, omics analyses are now commonplace in physiology research, where the whole point is to choose outcomes and make hypotheses after seeing the omics results. We are aware of the good intentions behind preregistration but again we see it as yet another bureaucratic chore that has gone too far and will prevent exploratory physiology. We find it thought provoking and a bit ironic that a journal with the name *Experimental Physiology* advocates practices that stifle the explorative elements in experimental physiological research. Perhaps it is time to update guidelines to accommodate modern methods and approaches to physiology research.

It is worth remembering that Christopher Columbus sailed into the undiscovered west in search of fortune and new worlds. He was driven by a vision – the courage to pursue an idea and the daring to venture into the unknown. Scientists in exploratory experimental physiology are driven by the same passion and should not be restrained by rigid prespecified analysis plans.

## AUTHOR CONTRIBUTIONS

All authors have read and approved the final version of this manuscript and agree to be accountable for all aspects of the work in ensuring that questions related to the accuracy or integrity of any part of the work are appropriately investigated and resolved. All persons designated as authors qualify for authorship, and all those who qualify for authorship are listed.

## CONFLICT OF INTEREST

None declared.

## FUNDING INFORMATION

None.
